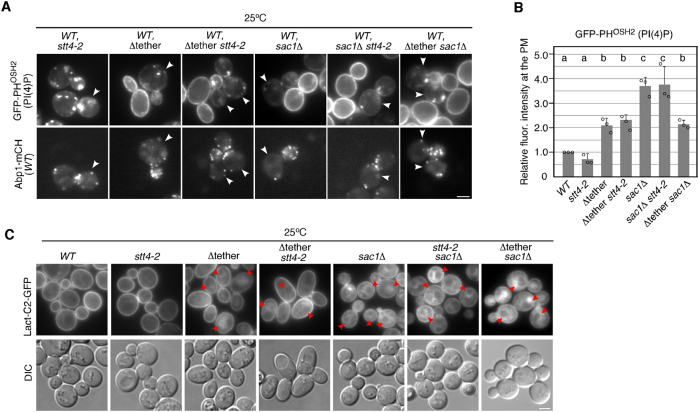# Maintenance of proper phosphatidylinositol-4-phosphate level by Stt4 and Sac1 contributes to vesicular transport to and from the plasma membrane

**DOI:** 10.1016/j.jbc.2025.110410

**Published:** 2025-06-21

**Authors:** Tomoki Sano, Makoto Nagano, Hiroki Shimamura, Wataru Yamamoto, Tomoyuki Tamada, Junko Y. Toshima, Jiro Toshima

**Affiliations:** 1 Department of Biological Science and Technology, Tokyo University of Science, Katsushika-ku, Tokyo, Japan; 2 School of Health Science, Tokyo University of Technology, Ota-ku, Tokyo, Japan

**Keywords:** phosphatidylinositol-4-phosphate, endocytosis, endoplasmic reticulum, plasma membrane, phosphatidylserine, secretion, recycling

## Abstract

Growing evidence suggests that counter-transport of phosphatidylinositol-4-phosphate (PtdIns(4)P) and phosphatidylserine (PS) at endoplasmic reticulum (ER)-plasma membrane (PM) contact sites is required for intracellular vesicle transport. PtdIns(4)P is metabolized by Stt4 PI 4-kinase residing at the PM and by Sac1 PtdIns(4)P phosphatase at the ER, and ER-PM contact sites are believed to be important for its efficient turnover. Recently, Stt4 has been shown to extensively localize to ER-PM contact sites. However, the precise location of Stt4 and the mechanism of localization to these sites have not been clarified. Additionally, although several studies have suggested a requirement for PS/PtdIns(4)P and sterol/PtdIns(4)P exchange at ER-PM contact sites in endocytosis, it is still unclear whether contact between the ER and the PM, turnover of PtdIns(4)P or PS, or maintenance of PtdIns(4)P or PS levels is more important. Here we found that Stt4 localizes to the cER regions where Scs2 and Ist2 are localized abundantly, and that localization of Stt4 is maintained in the Δtether mutant, which has a reduced number of ER-PM contact sites. We also demonstrated that the Δtether and *sac*1Δ mutants showed defects at different stages of endocytosis, and that the inactivation mutation of Stt4 restored the endocytosis defect only in the Δtether mutant. Furthermore, these mutants exhibited defective transport in the secretory and recycling pathways, and inactivation of Stt4 restored the secretory pathway in the Δtether mutant, but not the recycling pathway in either mutant. These results suggest that endocytosis, secretion, and recycling pathways are regulated directly or indirectly by different PtdIns(4)P-mediated mechanisms.

Clathrin-mediated endocytosis is regulated by more than 50 endocytic proteins that are assembled at the plasma membrane (PM) and then deform and invaginate the PM to form a clathrin-coated vesicle. These endocytic proteins are largely classified into three functional modules—the clathrin coat, actin and scission modules—based on their roles and the timing of their recruitment to the PM (1, 2). These modules are organized *via* endocytic protein–protein interactions or protein–lipid interactions. Clathrin adaptor proteins containing the lipid-binding domain contribute to anchorage of clathrin to the PM through interaction with anionic phospholipids, such as phosphatidylinositol-4,5-bisphosphate (PtdIns (4,5)P_2_) and phosphatidylserine (PS) (3, 4). PtdIns(4)P and PtdIns(4,5)P_2_ are generated by sequential phosphorylation of PtdIns by the PI kinases Stt4 and Mss4 at the PM (5, 6). Loss of the lipid-binding domain(s) of clathrin adaptor proteins or the *mss4* temperature-sensitive mutant causes significant endocytic defects, suggesting that interaction *via* the PtdIns(4, 5)P_2_ is critical for proper progression of the endocytosis process (3, 4, 7). In addition to clathrin adaptor recruitment, PtdIns(4, 5)P_2_ is also known to bind to the Bin-Amphiphysin-Rvs (BAR) domain protein and be implicated in membrane bending, invagination, and scission at later stages of endocytosis (8, 9, 10). In contrast to PtdIns(4,5)P_2_, the specific functions of PtdIns(4)P at the PM other than the biosynthetic precursor of PtdIns(4,5)P_2_ have not been established, although PtdIns(4)P plays essential roles in regulation of vesicle trafficking at the Golgi (11). In the previous study, we showed that localization of clathrin adaptor proteins, such as Ent1/Ent2 and Yap1801/Yap1802, at endocytic sites was decreased in cells where the level of PtdIns(4)P was decreased, whereas that of PtdIns(4,5)P_2_ was maintained (12), suggesting that PtdIns(4)P is also important for clathrin adaptor recruitment.

The level of PtdIns(4)P is regulated at endoplasmic reticulum (ER)-PM contact sites, where PM PtdIns(4)P is exchanged with PS or ergosterol in the cortical ER (cER), and dephosphorylated by the ER-associated PtdIns(4)P phosphatase, Sac1 (13, 14). Therefore, deletion of Sac1 or disruption of the ER-PM contact site causes a significant increase in the PtdIns(4)P level at the PM (15, 16). The ER also forms contacts with other organelles, including the Golgi, mitochondria, and endosomes, and these contact sites regulate lipid metabolism and transport (17, 18, 19). Previous studies have demonstrated that endocytic sites associate with the cER and that sterol transfer to the PM by the yeast oxysterol binding protein-related proteins (ORPs) Osh2 and Osh3 facilitates actin polymerization at endocytic sites (20, 21). Although several studies have suggested a requirement for PS/PtdIns(4)P and sterol/PtdIns(4)P exchanges at ER-PM contact sites in endocytosis (20, 21), it is not yet fully understood whether contact between the ER and the PM or maintenance of the PtdIns(4)P or PS level is more important. Most of the PM PtdIns(4)P in budding yeast is generated by the PI 4-kinase, Stt4 (22, 23, 24). Stt4 localizes to restricted regions of the PM known as PIK patches by forming a complex with Ypp1 and Efr3 (25, 26). Although PM PtdIns(4)P plays an important role in endocytosis, localization of PIK patches does not correspond to that of actin cortical patches, which are sites of endocytosis (25, 26). Unlike actin cortical patches, which show constant turnover at the PM, PIK patches are somewhat static structures (26). A recent study has demonstrated that Stt4-residing PIK patches localize extensively to ER-PM contact sites (27), but it has remained unclear where and how this occurs.

In the present study, we examined the requirement for anionic phospholipid transport *via* ER-PM contact sites in intracellular vesicle transport pathways, such as those involved in endocytosis, secretion, and endocytic recycling, in yeast. We demonstrated that the PI 4-kinase Stt4 localizes to the cER regions where Scs2 and Ist2 are localized abundantly. We also showed that localization of Stt4 was unaffected in the Δtether mutant, which has a reduced number of ER-PM contact sites. In the Δtether mutant, the levels of PM PtdIns(4)P and PtdIns(4, 5)P_2_ were markedly increased or decreased, respectively, whereas PS was mislocalized to the ER. Interestingly, the Δtether mutant exhibited defects in endocytosis and secretion, whereas these defects were suppressed by Stt4 loss of function mutation. In contrast, deletion of *SAC1* resulted in phenotypes similar to the Δtether mutant, but these phenotypes were unchanged by Stt4 inactivation. Additionally, these mutants exhibited defective transport in the recycling pathways, which was not restored by Stt4 inactivation. These results suggest that maintenance of PM PtdIns(4)P and/or PS at an appropriate level is important for these intracellular vesicle transport pathways.

## Results

### Stt4 is localized to the cER regions where Scs2 and Ist2 are localized abundantly

**Figure 1:**
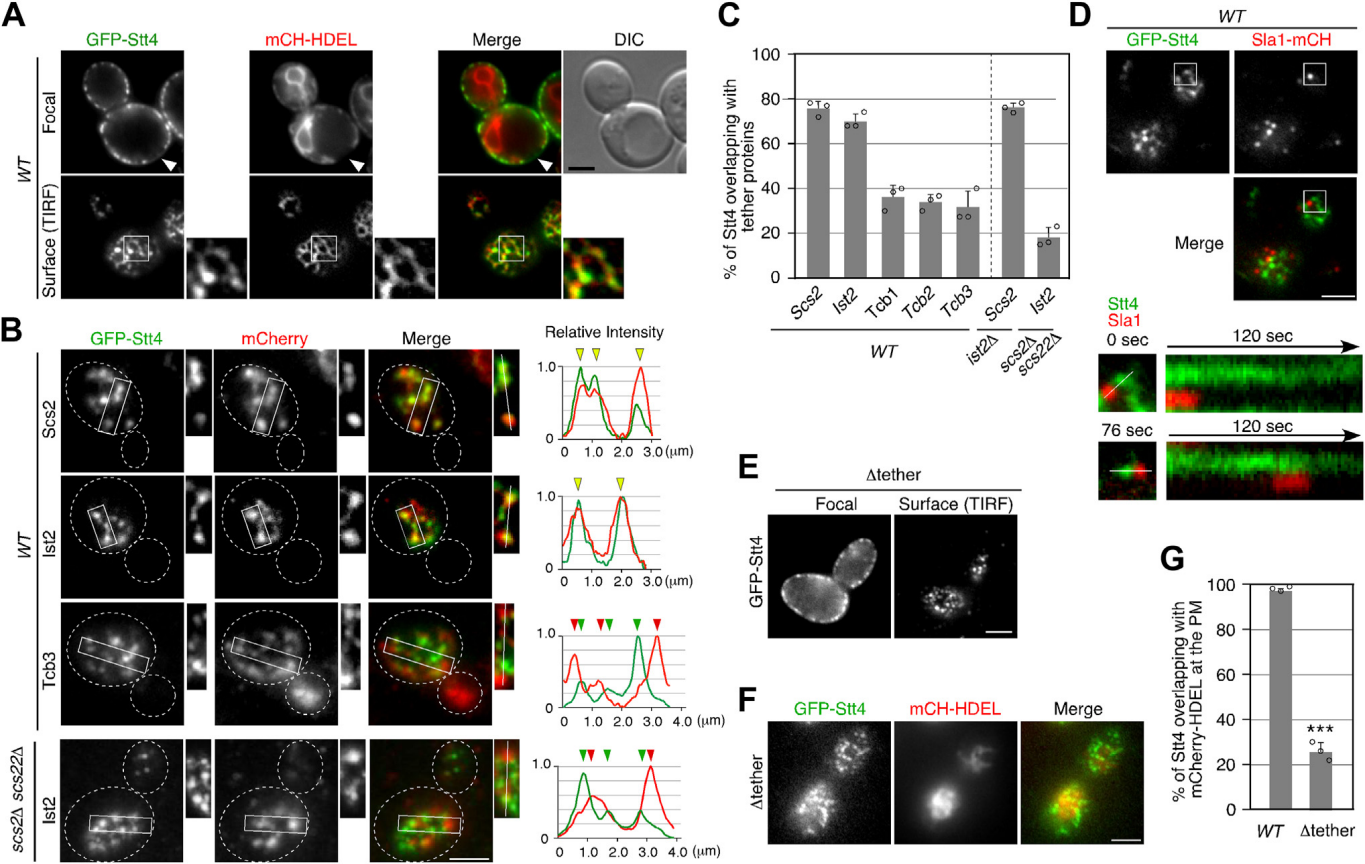
**Stt4 is localized to the restricted ER-PM contact sites**. *A*, localization of GFP-Stt4 visualized at medial focal plane or surface of wild-type cell. Cells expressing GFP-Stt4 and mCherry-HDEL were grown to early to mid-logarithmic phase in YPD medium at 25 ^o^C and observed by fluorescence microscopy and different interference contrast (DIC). *B*, localization of GFP-Stt4 and mCherry-tagged tethering proteins in wild-type cells. Each image pair was acquired simultaneously using dual-channel imaging system (see METHODS for details). Representative fluorescence intensity profiles along a line in the boxed areas are shown to the *right*. *C*, quantification of GFP-Stt4 overlapping with mCherry-tagged proteins. Data show the mean ± SEM from n ≥ 3 experiments (n > 30 puncta for each experiment). *D*, localizations of GFP-Stt4 at a surface of wild-type cell. Kymographs along line in the boxed area are shown in the lower panels. *E*, localization of GFP-Stt4 visualized at medial focal plane or surface of the Δtether mutant. *F*, localizations of GFP-Stt4 at a surface of the Δtether mutant. *G*, quantification of GFP-Stt4 overlapping with mCherry-HDEL in the Δtether mutant. Data show the mean ± SEM from n ≥ 3 experiments (n > 30 puncta for each experiment). ∗∗∗, *p* value < 0.001, two-tailed unpaired *t* test with Welch’s correction. The surface localization of all fluorescent proteins was observed by TIRFM. Scale bars, 2.5 μm.

**Figure S1:**
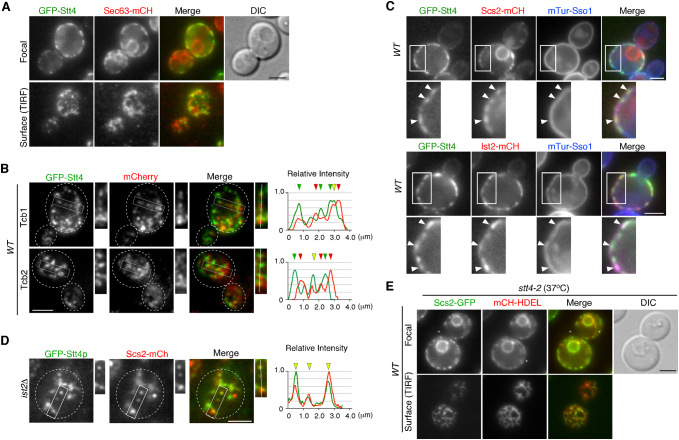


A previous study has demonstrated that the localization of Stt4 PIK patches extensively overlaps that of the cER (27), although the precise localization of Stt4 in the cER has yet to be determined. To clarify the localization of Stt4, we utilized an N-terminal GFP-tagged protein expressed from the endogenous locus. Consistent with the previous study, we observed that GFP-Stt4 was localized at the cER labeled by mCherry protein containing an ER retention signal (mCherry-HDEL), although the strong signals of GFP-Stt4 did not completely overlap the localization of mCherry-HDEL (Fig. 1*A*, upper panels). To investigate the precise location of GFP-Stt4 at the PM, we imaged GFP-Stt4 and mCherry-HDEL using total internal reflection fluorescence microscopy (TIRFM). Interestingly, we found that GFP-Stt4 did not entirely localize at ER but was localized more to restricted regions at the contact sites (Fig. 1*A*, lower panels). We also compared the localization of GFP-Stt4 with chromosomally mCherry-tagged Sec63, an ER integral membrane protein that is ubiquitously localized in the ER membrane and found that Stt4 is localized to restricted regions in the ER labeled with mCherry-Sec63 (Fig. S1*A*). We next examined the localization of GFP-Stt4 using mCherry-tagged ER-PM tethering proteins. It has been demonstrated that tethering proteins, such as the VAP (VAMP-associated protein) family protein Scs2, the tricalbin family Tcb1/Tcb2/Tcb3, and the TMEM16 homologue Ist2, are distributed non-homogeneously with the cER (28). A previous study demonstrated that Tcb1, Tcb2, and Tcb3 exhibit exclusive localization to the cER, and that the distribution of Tcb1 and Tcb3 mostly overlaps (16, 28). In contrast, the localization of Tcb3 and Scs2 or Ist2 does not overlap completely (28). The localization of another VAP family protein, Scs22, has not been sufficiently characterized in comparison to Scs2. We analyzed the degree of overlap by plotting the fluorescence intensity profile of each protein and found that paired profiles of GFP-Stt4 and Scs2-mCherry, as well as GFP-Stt4 and Ist2-mCherry, overlapped extensively (Fig. 1*B*). In contrast, the intensity profiles of GFP-Stt4 and Tcb3-mCherry did not completely overlap. Consistent with this result, quantitative analyses revealed that GFP-Stt4 was highly colocalized with mCherry-tagged Scs2 or Ist2 (∼75.7% or ∼70.0%), relative to the tricalbin family (∼37.9, ∼33.9, or ∼31.5%, respectively) (Figs. 1*B*, *C*, and S1*B*). These observations indicated that Stt4 was localized to the cER regions where Scs2 and Ist2 were abundant. Also, by using mTurquoise (mTur)-Sso1, a marker for the PM, we confirmed that the regions of overlap between Stt4 and Scs2 or Ist2 are at the ER-PM contact sites (Fig. S1*C*). A previous study has demonstrated that Stt4 PIK patch subunit Efr3 directly interacts with Scs2 through interaction between the MSP domain of Scs2 and a FFAT motif in Efr3 (27). We next investigated the effect of deletion of Scs2/Scs22 or Ist2 on the localization of Stt4. The overlap between GFP-Stt4 and Ist2-mCherry signals was significantly decreased in *scs2*Δ *scs22*Δ cells (∼18.0%), whereas the overlap between GFP-Stt4 and Scs2-mCherry signals remained largely unchanged in *ist2*Δ cells (∼76.1%) (Figs. 1*B*, *C*, and S1*D*). Thus, Scs2/Scs22 seems to play an important role in the localization of Stt4 at ER-PM contact sites. probably through the interaction with Efr3. We next compared the localization and dynamics of GFP-Stt4 patches with those of Sla1 patches, which are sites of endocytosis, using TIRFM. GFP-Stt4 and Sla1-mCherry rarely colocalized and showed quite different dynamics: Sla1 appeared at the PM, remained localized there for about 30 to 40 s, and then disappeared, whereas Stt4 was localized persistently at the PM (Fig. 1*D*).

We then sought to examine the requirement of ER-PM contact sites for Stt4 localization and generated a Δtether mutant lacking all of six proteins (Ist2, Scs2/Scs22, Tcb1/Tcb2/Tcb3) that tether the PM to the ER (16). To assess the resulting defect at ER-PM contact sites, we expressed mCherry-HDEL with the Δtether mutant and observed the localization of the cER. As shown in the previous studies (16), the cER forms extensive contacts with the PM in wild-type cells, whereas in the Δtether mutant the contact sites are remarkably reduced (Fig. 1*F*). Interestingly, the Δtether mutant exhibited Stt4 localization similar to that in wild-type cells (Fig. 1*E*), although GFP-Stt4 signals overlapping with mCherry-HDEL were significantly decreased (∼25.7%) (Fig. 1, *F* and *G*). These results indicated that Stt4 localization in puncta at the PM is independent of formation of the ER-PM contact sites.

### Loss-of-function mutation in the STT4 gene reduces the increase in PtdIns(4)P level at the PM in Δtether and sac1Δ mutants

**Figure 2:**
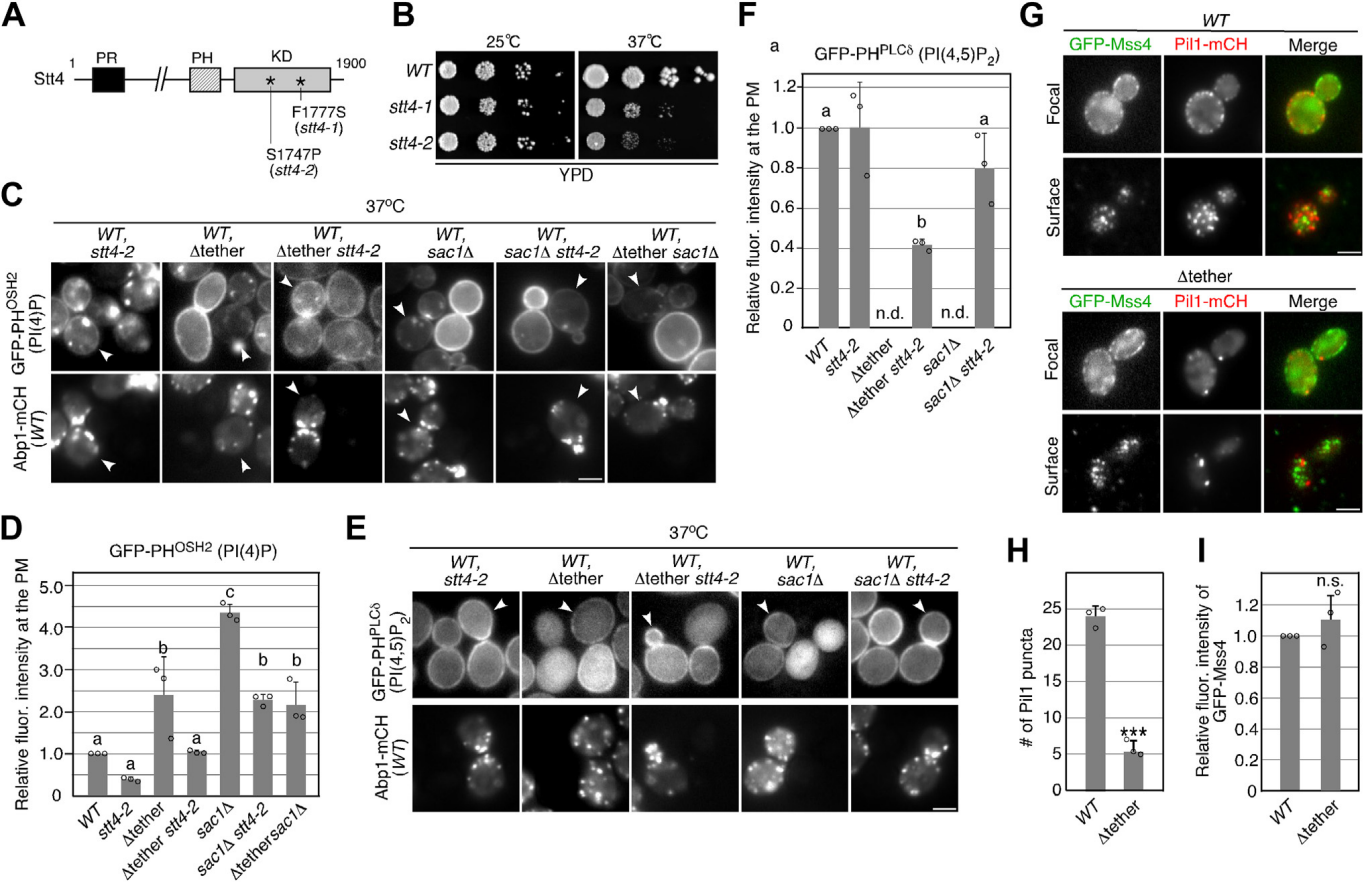
**The *stt4-2* mutation restores increased PtdIns**(**4**)**P and decreased PtdIns**(**4,5**)**P_2_ levels in the Δtether and *sac1*Δ mutants**. *A*, structure of Stt4 mutants. Two mutation sites are indicated. *B*, plates showing the growth phenotype of *stt4-1* and *stt4-2* mutants. A dilution series of the indicated cells were plated on YPD plates and incubated at 25 ^o^C or 37 ^o^C to compare cell growth. *C* and *E*, Localization of GFP-PH^Osh2^ (*C*) or GFP-PH^PLCδ^ (*E*) in wild-type and mutant cells. The mutant cells expressing GFP-PH^Osh2^ or GFP-PH^PLCδ^ and wild-type cells expressing Abp1-mCherry and GFP-PH^Osh2^ or GFP-PH^PLCδ^ were each grown to early to mid-logarithmic phase in YPD medium at 37 ^o^C for 2 h. Subsequently, approximately the same number of wild-type and mutant cells were mixed and acquired in the same images. Only wild-type cells were labeled by Abp1-mCherry in the lower images. Arrowheads indicate wild-type cells. N.D.: not-detected. *D* and *F*, quantification of the fluorescence intensity of GFP-PH^Osh2^ or GFP-PH^PLCδ^ in wild-type and mutant cells. Relative fluorescence intensity of GFP-PH^Osh2^ or GFP-PH^PLCδ^ was calculated as described in the Methods. Data show mean ± SEM from at least three experiments, with >30 cells counted for each strain per experiment. Different letters indicate significant differences at *p* < 0.05 (*i*.*e*., no significant difference for a vs. a, significant difference for a vs. b with *p* < 0.05), one-way ANOVA with Tukey’s *post hoc* test. *G*, localization of GFP-Mss4 and Pil1-mCherry visualized at medial focal plane or surface of wild-type or Δtether cell. The surface localization was observed by TIRFM. *H*, Quantification of the number of GFP-Mss4 puncta displayed in (*G*). Data show mean ± SEM from at least three experiments, with >30 cells counted for each strain per experiment. *I*, relative fluorescence intensity of GFP-Mss4 puncta in wild-type and Δtether cell. Data show mean ± SEM from at least three experiments, with >30 cells counted for each strain per experiment. ∗∗∗, *p* value < 0.001, two-tailed unpaired *t* test with Welch’s correction. ns: non-statistically significance. Scale bars, 2.5 μm.

To explore the role of Stt4 at ER-PM contact sites, we utilized a temperature-sensitive (ts) mutant for *STT4*. In the previous study, we isolated the *stt4-1* mutant, which has a Phe-to-Ser mutation at amino acid 1777 (Fig. 2*A*), by PCR-based random mutagenesis of the catalytic domain of the kinase and characterized it (12). Using the same method, we succeeded in isolating another ts allele of *STT4* (*stt4-2*) with a Ser-to-Pro mutation at position 1747, creating a more severe growth defect phenotype at 37 ^o^C than that of the *stt4-1* mutant (Fig. 2, *A* and *B*). We first examined the localization of Scs2 at the cER in the *stt4-2* mutant at 37 ^o^C and found that the localization was largely unaffected (Fig. S1*E*). We then examined the localization of PtdIns(4)P at the PM and Golgi in the *stt4-2* cells. PtdIns(4)P levels at the PM were determined by measuring the intensity of GFP-PH^Osh2^, a specific fluorescence probe for PtdIns(4)P (29, 30). To precisely evaluate differences in the fluorescence intensity of the probe, the *stt4-2* mutant was compared directly alongside wild-type cells (Fig. 2*C*). In the *stt4-2* mutant, the PM localization of the PtdIns(4)P binding protein GFP-PH^Osh2^ was decreased to ∼39.0% of that in wild-type cells at 37 ^o^C, whereas it exhibited similar localization to that in wild-type cells at 25 ^o^C (Figs. 2*C*, *D*, S2, *A* and *B*). In contrast, the PM localization of PtdIns(4)P was increased ∼2.1 fold at 25 ^o^C and ∼2.4 fold at 37 ^o^C in the Δtether mutant (Figs. 2*C*, *D*, S2, *A* and *B*). Interestingly, the increased PtdIns(4)P level in the Δtether mutant was decreased to a level comparable to that in wild-type cells only at 37 ^o^C in the Δtether *stt4-2* double mutant (Figs. 2*C*, *D*, S2, *A* and *B*). On the other hand, in cells lacking the *SAC1* gene, the PtdIns(4)P level was increased ∼1.8 fold at both 25 ^o^C and 37 ^o^C, relative to that in the Δtether mutant cells (Figs. 2*C*, *D*, S2, *A* and *B*). In the Δtether *sac1*Δ double mutant, the PtdIns(4)P level was equivalent to that in the Δtether mutant at 37 ^o^C (Figs. 2*C*, *D*, S2, *A* and *B*). The finding that deletion of Sac1 has a more severe effect on the level of PM PtdIns(4)P compared to the Δtether mutant suggests that the dephosphorylation of PM PtdIns(4)P by Sac1 is regulated by pathways other than exchange transport at the PM-ER contact site. Although the increased PtdIns(4)P level in the *sac1*Δ mutant was decreased to around half in the *stt4-2 sac1*Δ double mutant at 37 ^o^C, it was ∼2.3 fold higher than that in wild-type cells. This observation suggests that PI 4-kinases other than Stt4, possibly Golgi-resident Pik1, may increase the level of PM PtdIns(4)P through vesicle transport.

We next assessed the PtdIns(4,5)P_2_ levels at the PM in these mutants by measuring the intensity of GFP-PH^PLCδ^, a specific fluorescence probe for PtdIns(4,5)P_2_ (31). Similarly to our previous observation (12), the *stt4-2* mutant showed little effect on the PtdIns(4,5)P_2_ level at the PM (Fig. 2, *E* and *F*). Unexpectedly, in the Δtether or *sac1*Δ mutant, GFP-PH^PLCδ^ was diffusely evident in the cytosol, and barely detectable at the PM (Fig. 2, *E* and *F*). The decreased PtdIns(4,5)P_2_ level at the PM was confirmed by localization of mCherry-fused Pil1, which is an eisosome protein exhibiting PtdIns(4,5)P_2_-dependent localization (32). As shown previous study, Pil1-mCherry demonstrated multiple localization of puncta (eisosome localization) at the PM in wild-type cells (∼24.0 puncta/cell), whereas in the Δtether mutant the number of Pil1-mCherry puncta were significantly decreased (∼5.3 puncta/cell) (Fig. 2, *G* and *H*). The localization of Pil1 in the Δtether mutant resembled that observed in cells depleted of PtdIns (4,5)P_2_ (32). In contrast, the localization of PtdIns(4)P 5-kinase Mss4 was not significantly affected in the Δtether mutant (Fig. 2*I*), suggesting that Mss4 function might be suppressed. The decreased level of PtdIns(4,5)P_2_ was restored by additional *stt4-2* mutation to these mutants (∼41.9% or ∼80.2%, respectively), as was the case for the PtdIns(4)P level (Fig.2, *E* and *F*). Thus, dephosphorylation of PtdIns(4)P *via* ER-PM contact sites seem to be crucial for production of PtdIns(4,5)P_2_ at the PM.

### Requirement of ER-PM contact sites for endocytic internalization

**Figure 3:**
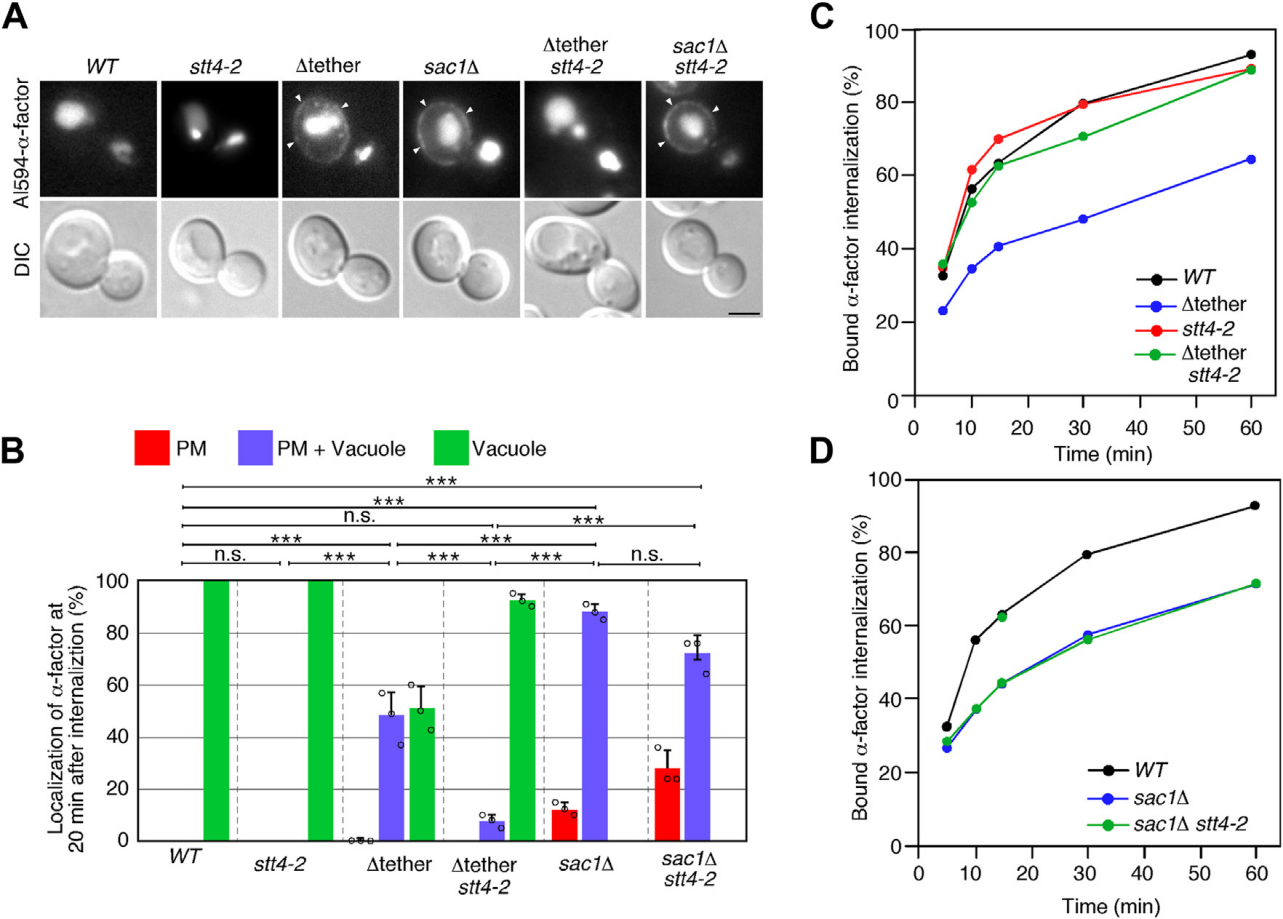
**The *stt4-2* mutation restores the defect in endocytosis in the Δtether mutant but does not restore in *sac1*Δ mutants**. *A*, defective transport of A594-α-factor in the Δtether and *sac1*Δ mutants. Wild-type and mutant cells were grown to early to mid-logarithmic phase in YPD medium at 25 ^o^C and further cultured 37 ^o^C for 2 h, and treated with A594-α-factor, and the images were acquired 30 min after washing out unbound A594-α-factor and warming the cells to 37 °C. Arrowheads indicate localization of A594-α-factor at the PM. *B*, quantification of localization of A594-α-factor in wild-type and mutant cells. The bar graphs represent the percentages of cells exhibiting A594-α-factor localized at PM only (*red*), PM and vacuole (*blue*), or vacuole only (*green*) at 30 min after internalization. Data show mean ± standard deviation (SD) from three experiments, with 50 cells counted for each strain per experiment. ∗∗∗, *p* value < 0.001, two-tailed unpaired *t* test with Welch’s correction. ns: non-statistically significant. *C* and *D*, radiolabeled α-factor internalization assays were performed on the indicated strains at 37 °C. Each curve represents the average of two independent experiments. Scale bars, 2.5 μm.

Previous studies have demonstrated that a portion of the cER forms contacts with endocytic sites and plays a role in facilitating actin-mediated membrane invagination for endocytic internalization using the *rtn1*Δ reticulon mutant, in which the cER tubules collapse to a lamina (21). However, since in the reticulon mutant the ER-PM contact sites are not completely lost, we speculate that the Δtether mutant exhibits a more prominent defect of endocytic internalization. Additionally, several studies suggested that the *sac1*Δ mutant exhibits defects in late steps of endocytic pathway, such as endosomal trafficking and vacuolar formation (33, 34, 35), but it has not been clarified exactly which step(s) is defective. To accurately examine the efficiency of endocytosis in these mutants, we utilized three different endocytic markers: fluorescent α-factor, which can visualize transport of cargo from the PM to the vacuole, ^35^S-labeled α-factor, which can monitor endocytic cargo internalization, and Sla1-GFP and Abp1-mCherry, which can visualize the formation and internalization of clathrin-coated vesicles. As reported previously, when added to wild-type cells, A594-α-factor was transported to the vacuole within 20 min (Fig. 3*A*) (36). In the Δtether mutant, A594-α-factor was still partially localized to the PM at 20 min after internalization, indicating that this mutant has a defect of α-factor internalization. In contrast, the *stt4-2* mutant showed little effect on both α-factor internalization and transport to the vacuole, and the defect observed in the Δtether mutant was considerably ameliorated in the Δtether *stt4-2* double mutant (Fig. 3*A*). Interestingly, the *sac1*Δ mutant exhibited a defect similar to that of the Δtether mutant, but the defect was not ameliorated in the *sac1*Δ *stt4-2* double mutant (Fig. 3*A*). Quantitative analysis categorizing A594-α-factor localization as PM, PM and vacuole, or vacuole-only revealed that the Δtether mutants had an obvious defect of A594-α-factor transport (PM and vacuole: ∼48.3%), which was abrogated when combined with the *stt4-2* mutant (∼7.7%) (Fig. 3*B*). The *sac1*Δ mutant showed more severe defect (∼88.0%), and this defect was not significantly abrogated in the *sac1*Δ *stt4-2* double mutant (Fig. 3*B*). We next examined the effect on endocytosis by assessing the internalization of ^35^S-labeled α-factor. As shown in Figure 3*C*, both the Δtether and *sac1*Δ mutants exhibited a defect of α-factor internalization. Consistent with the analysis using A594-α-factor, recovery of ^35^S-labeled α-factor internalization was observed only in the Δtether *stt4-2* mutant, and not in the *sac1*Δ *stt4-2* mutant (Fig. 3, *C* and *D*). These results also correlate with the levels of phosphoinositides at the PM, suggesting that the effects on endocytic internalization are associated with an increased level of PtdIns(4)P or a decreased level of PtdIns(4,5)P_2_.

### Δ*tether* and sac1Δ mutants have different effects on endocytic vesicle formation and internalization

**Figure 4:**
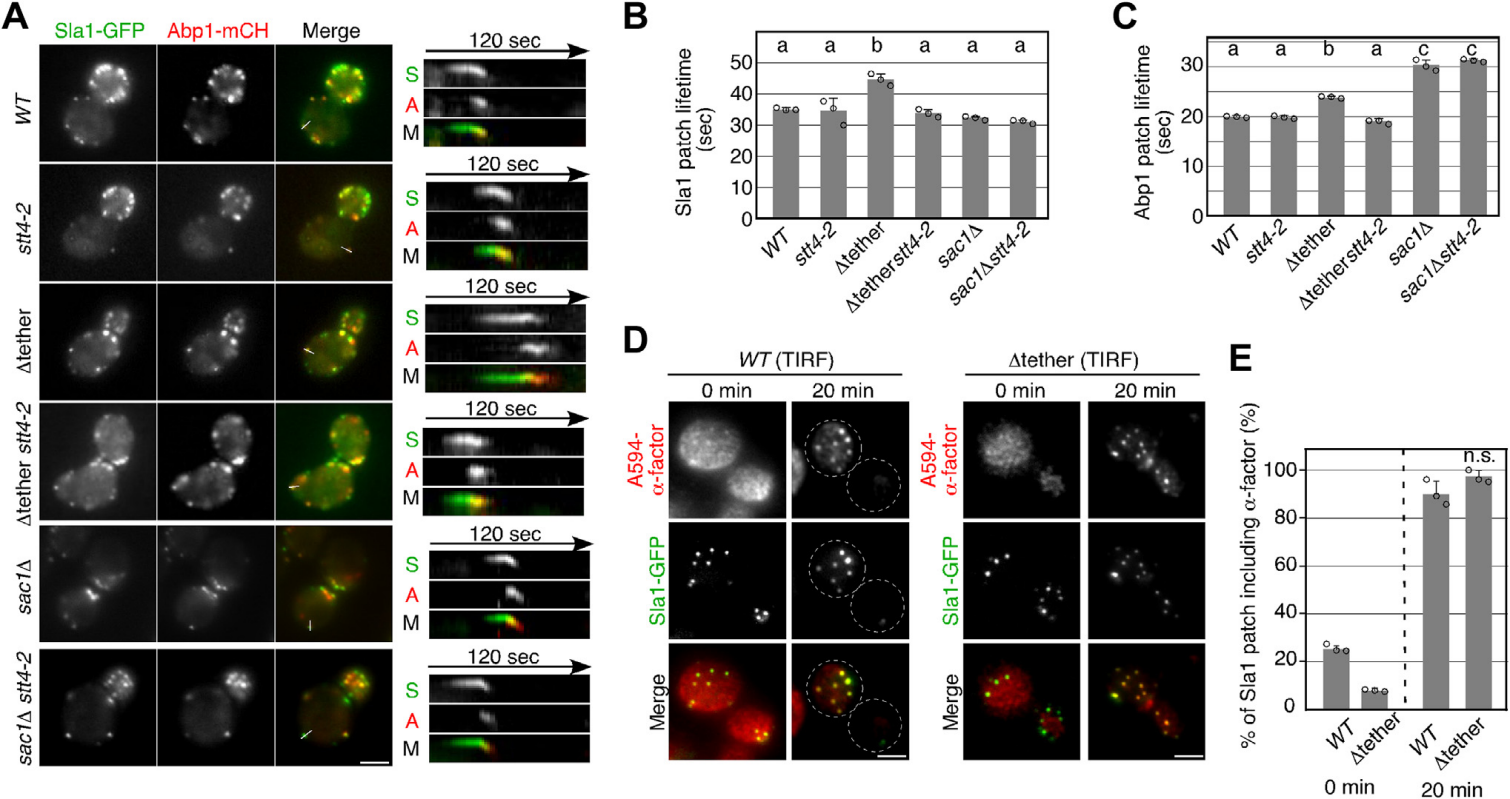
**The Δtether and *sac1*Δ mutants show defects at different stages of the formation of clathrin-coated vesicles**. *A*, Localizations of Sla1-GFP and Abp1-mCherry in wild-type and mutant cells. Cells expressing Sla1-GFP and Abp1-mCherry were grown to early to mid-logarithmic phase in YPD medium at 25 ^o^C and further cultured 37 ^o^C for 2 h, and observed by fluorescence microscopy. Kymographs along lines in the merged images are shown in the *right panels*. *B* and *C*, average lifetimes of Sla1-GFP patches (*B*) or Abp1-mCherry patches (*C*) ± SD for indicated strains. *n* = 50 patches for each strain. Different letters indicate significant differences at *p* < 0.05, one-way ANOVA with Tukey’s *post hoc* test. *D*, localization of A594-α-factor and Sla1-GFP in wild-type and Δtether mutant cells treated with LatA. After incubating cells expressing Sla1-GFP with 200 μM LatA at 25 °C for 30 min, they were labeled with A594-α-factor in the presence of LatA. The images were acquired at 0 or 20 min after washing out unbound A594-α-factor and warming the cells to 25 ^o^C and incubating them with glucose-containing medium in the continuous presence of 200 μM LatA. *E*, quantification of co-localization of A594-α-factor and Sla1-GFP in individual cells. *Error bars* represent the SEM from at least three experiments. n.s.: non-statistically significant, one-way ANOVA with Tukey’s test. Scale bars, 2.5 μm.

Given the reduced internalization of α-factor from the PM, we next investigated the effects of these mutations on the formation and internalization of endocytic vesicles. We used Sla1-GFP, a marker of the clathrin late coat, and Abp1-mCherry, a marker of membrane invagination, to follow the dynamics of endocytic vesicles (Fig. 4*A*) (37). First, we carried out simultaneous imaging of Sla1-GFP and Abp1-mCherry to analyze the dynamic behavior of these proteins in live cells. In wild-type cells, Sla1-GFP first appeared at cortical patches, then Abp1-mCherry was recruited, and internalized together into the cytosol, as shown in kymographs generated across a single-pixel-wide line for an individual patch (Fig. 4*A*). The mean lifetimes of Sla1-GFP and Abp1-mCherry patches in wild-type cells were ∼35.1 s and ∼19.9 s, respectively (Fig. 4, *B* and *C*). Although previous studies reported that *stt4* ts mutant failed to appropriately organize the actin cytoskeleton at restrictive temperature (24, 35), we could not detect any defects in localization or dynamics of cortical patches in the *stt4-2* mutant (Fig. 4, *A*–*C*). Interestingly, the Δtether and *sac*1Δ mutants differentially affected the dynamics of Sla1-GFP and Abp1-mCherry patches. In the Δtether mutant, the lifetime of the Sla1-GFP patch was increased (∼44.5 s), and it was normalized when combined with the *stt4-2* mutant (∼33.8 s) (Fig. 4, *A* and *B*). The dynamics and lifetime of Abp1-mCherry patch was slightly increased (∼23.9 s) in comparison to wild-type cells, and it was also recovered by additional *stt4-2* mutation (Fig. 4, *A* and *C*). In the *sac*1Δ mutant, on the other hand, the lifetime of the Abp1-mCherry patch was apparently increased (∼30.3 s) whereas that of Sla1 was not affected (∼32.1 s), and the increased lifetime was not normalized in the *sac*1Δ *stt4-2* mutant (∼31.33 s) (Fig. 4, *A*–*C*). We have previously demonstrated that PtdIns(4)P is involved in ligand-induced receptor recruitment to clathrin-coated pits (12). Accordingly, we examined the dynamics of A594-α-factor bound to cell surface receptors in the Δtether mutant, but no apparent difference in A594-α-factor recruitment to clathrin-coated pits was observed between wild-type and Δtether mutant cells (∼90.2% and ∼97.2%) (Fig. 4, *D*–*F*). These results suggest that the Δtether and *sac*1Δ mutants have defects at different stages of endocytosis: the former shows defective clathrin coat formation, whereas the latter shows defective vesicle internalization.

### Δ*tether* and sac1Δ mutants show accumulation of phosphatidylserine to the ER membrane and cause defects in the secretory and recycling pathway

**Figure 5:**
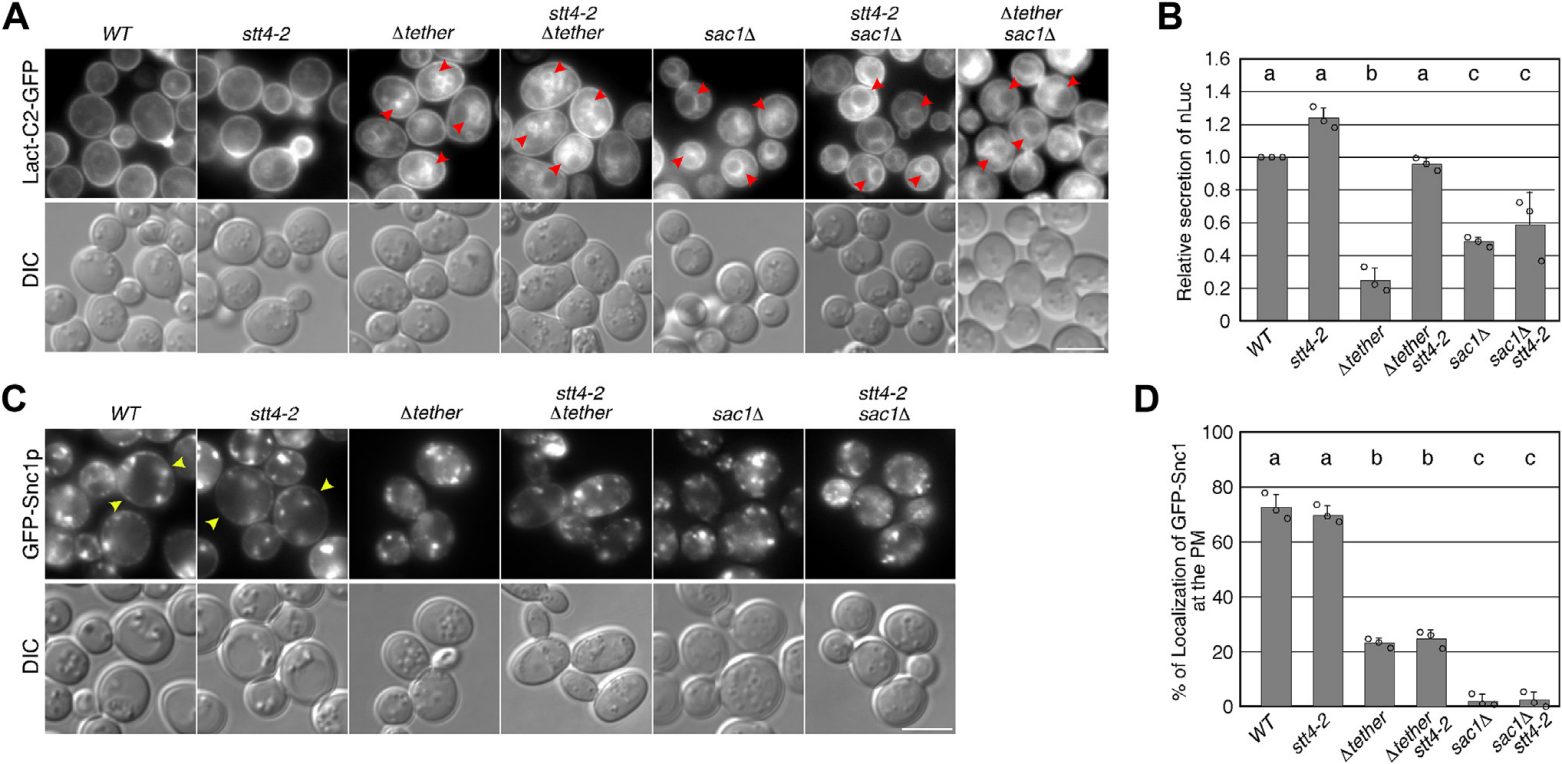
**Localization of PS in the Δtether and *sac1*Δ mutants and its effect on the secretion and recycling pathway**. *A*, localization of PS in wild-type and mutant cells. Cells expressing Lact-C2-GFP were grown to early to mid-logarithmic phase in YPD medium at 25 ^o^C and further cultured 37 ^o^C for 2 h, and observed by fluorescence microscopy and differential interference contrast (DIC). *Red* arrowheads indicate aberrant PS accumulation in the intracellular compartments. *B*, data are shown as relative value of luciferase activity in culture media of wild-type cell. NanoLuc luciferase-based secretion assays were performed on the indicated strains at 37 ^o^C. Data show mean ± SD from at least three experiments. Different letters indicate significant differences at *p* < 0.05, one-way ANOVA with Tukey’s *post hoc* test. *C*, localization of GFP-Snc1 in wild-type and mutant cells. Cells expressing GFP-Snc1 were grown to early to mid-logarithmic phase in YPD medium at 25 ^o^C and further cultured 37 ^o^C for 2 h, and observed by fluorescence microscopy and DIC. Yellow arrowheads indicate GFP-Snc1 localized at the PM. *D*, quantification of localization of GFP-Snc1 at the plasma membrane (PM). The localization of GFP-Snc1 at the PM was quantified as the percentage of cells in which the fluorescent intensity of Snc1-GFP at the PM is higher than that in the cytosol. Data show mean ± SEM from at least three experiments, with >30 cells counted for each strain per experiment. Different letters indicate significant differences at *p* < 0.05, one-way ANOVA with Tukey’s *post hoc* test. Scale bar, 5.0 μm.

Previous studies have suggested that PtdIns(4)P is a key factor for counter-transport of PS *via* ER-PM contact sites (38). We previously demonstrated that in cells lacking the PS synthase Cho1, the processes of endocytosis were mostly unaffected, whereas protein transport from the *trans*-Golgi network (TGN) to the PM was severely impaired (39). Additionally, recent study reported that the tether and *sac1*Δ mutants exhibit decreases in PS at the PM (40). Therefore, we investigated the effect of Δtether or *sac1*Δ mutation on the localization of PS and the effects of *stt4-2* mutation in these mutants. To observe PS localization, we utilized the GFP-fused C2 domain of bovine lactadherin (Lact-C2-GFP) (41, 42). Consistent with previous studies (43), we observed that Lact-C2-GFP signals were exclusively localized at the PM in wild-type cells (Fig. 5*A*). The *stt4-2* mutant exhibited similar localization to that in wild-type cells, whereas aberrant accumulation of GFP-Lact-C2 to the intracellular compartments was evident in the Δtether and *sac1*Δ mutants at both 25 ^o^C and 37 ^o^C (Figs. 5*A* and S2*C*), as shown previously (40). In contrast to the effect on PtdIns(4)P and endocytosis, this aberrant PS localization was not normalized in either the Δtether *stt4-2* or the *sac1*Δ *stt4-2* mutant (Figs. 5*A* and S2*C*). This result prompted us to further examine the effect of Δtether or *sac1*Δ mutation on the secretory and recycling pathway, which includes the transport route from the TGN to the PM, using a NanoLuc luciferase reporter containing an N-terminal secretion signal (secNluc) or GFP-Snc1 as a marker for each pathway (39). To quantify the amount of protein secretion from cells, the secNluc gene was chromosomally integrated into both wild-type and mutant cells, and the activity of secNluc secreted into the culture medium was assessed. Consistent with the previous studies (24, 44), no apparent defect was observed in Nluc secretion in the *stt4-2* mutant (Fig. 5*B*). Interestingly, the luciferase activity in the culture medium of the Δtether and *sac1*Δ mutants was significantly decreased (∼24.8% and ∼48.4%), compared to that of wild-type cells (Fig. 5*B*), and the decrease in activity was normalized only in the Δtether *stt4-2* mutant (∼96.2%) (Fig. 5*B*). We then examined the effect on trafficking of GFP-Snc1, an exocytic v-SNARE that is endocytosed, transiently localized to the early/sorting compartment at the TGN, and then recycled back to the PM (45, 46). In wild-type cells, GFP-Snc1 was localized at the PM with some punctate staining of internal structures (Fig. 5*C*), as shown in previous studies (45, 47). In the Δtether or *sac1*Δ mutant, localization of GFP-Snc1 was shifted to intracellular compartments (Fig. 5*C*). Quantitative analysis showed that the proportion of cells exhibiting GFP-Snc1 localization at the PM was decreased to ∼23.3% or ∼2.0% for the Δtether or *sac1*Δ mutant, respectively, relative to that of wild-type cells (Fig. 5*D*). Intriguingly, in contrast to the effects on endocytosis or the secretory pathway, the PM localization of GFP-Snc1 was not normalized in either the Δtether or the *sac1*Δ mutant (Fig. 5, *C* and *D*). These results suggest that maintenance of an appropriate level of PtdIns(4)P is important in the secretory pathway, whereas counter-transport of PS to the PM is important in the recycling pathway.

## Discussion

Physical contact between the cER and the PM is mediated by several ER-integral membrane proteins that interact with the cytoplasmic face of the PM (16). These ER-PM tethering proteins are distributed non-homogeneously within the cER; the distribution of Scs2 and Tcb3, as well as that of Ist2 and Tcb3, does not overlap completely, whereas that of Tcb1 and Tcb3 does overlap completely (28). The distribution of these tethering proteins correlates with differences in cER shape; Scs2 and Ist2 are distributed predominantly at flat cER sheets, whereas Tcb3 localizes predominantly to tubular cER and the curved edges of cER sheets (28). In this study we showed that Stt4 was localized at the cER regions where Scs2 and Ist2 were localized abundantly, suggesting that Stt4 also localizes to the cER sheets. A recent study reported that >80% of Stt4 PIK patches were localized at cER sheets, and that this localization was not affected in cells lacking reticulon proteins (Rtn1, Rtn2, and Yop1), which shape the ER network into highly curved tubules (27). It was also shown that the PIK patch subunit Efr3 interacts with Scs2 (27). These observations are consistent with our finding that Stt4 was highly colocalized with Scs2 and Ist2, but not with Tcb3. Interestingly, we found that puncta localization of Stt4 at the PM was unaffected in the Δtether mutant. Thus, the Stt4 appears to localize independently of the formation of ER-PM contact sites.

The significance of Stt4 localization at ER-PM contact sites is not well understood, but by localizing there Stt4 could efficiently control PtdIns(4)P levels not only at the PM but also at the membrane of other organelles, such as mitochondria and endosomes. Previous studies have shown that ER-mitochondria and ER-endosome contact sites function as a platform for phosphatidylethanolamine (PE) and phosphatidylcholine (PC) production (48, 49). Through these contact sites PS is transferred to mitochondria or endosomes, where it is converted to PE by PS decarboxylase Psd1 or Psd2. PE is then returned to the ER for completion of PC synthesis (48). This Psd2-dependent PS decarboxylation requires specialized ER-endosomal contact sites assembled by a complex including Psd2 and Stt4 (48). Since the activity of Stt4 is required for sufficient activation of Psd2 in the endosome (50), localization of Stt4 at the ER-PM contact site might be necessary for efficient conversion of PS to PE in the endosome.

**Figure 6:**
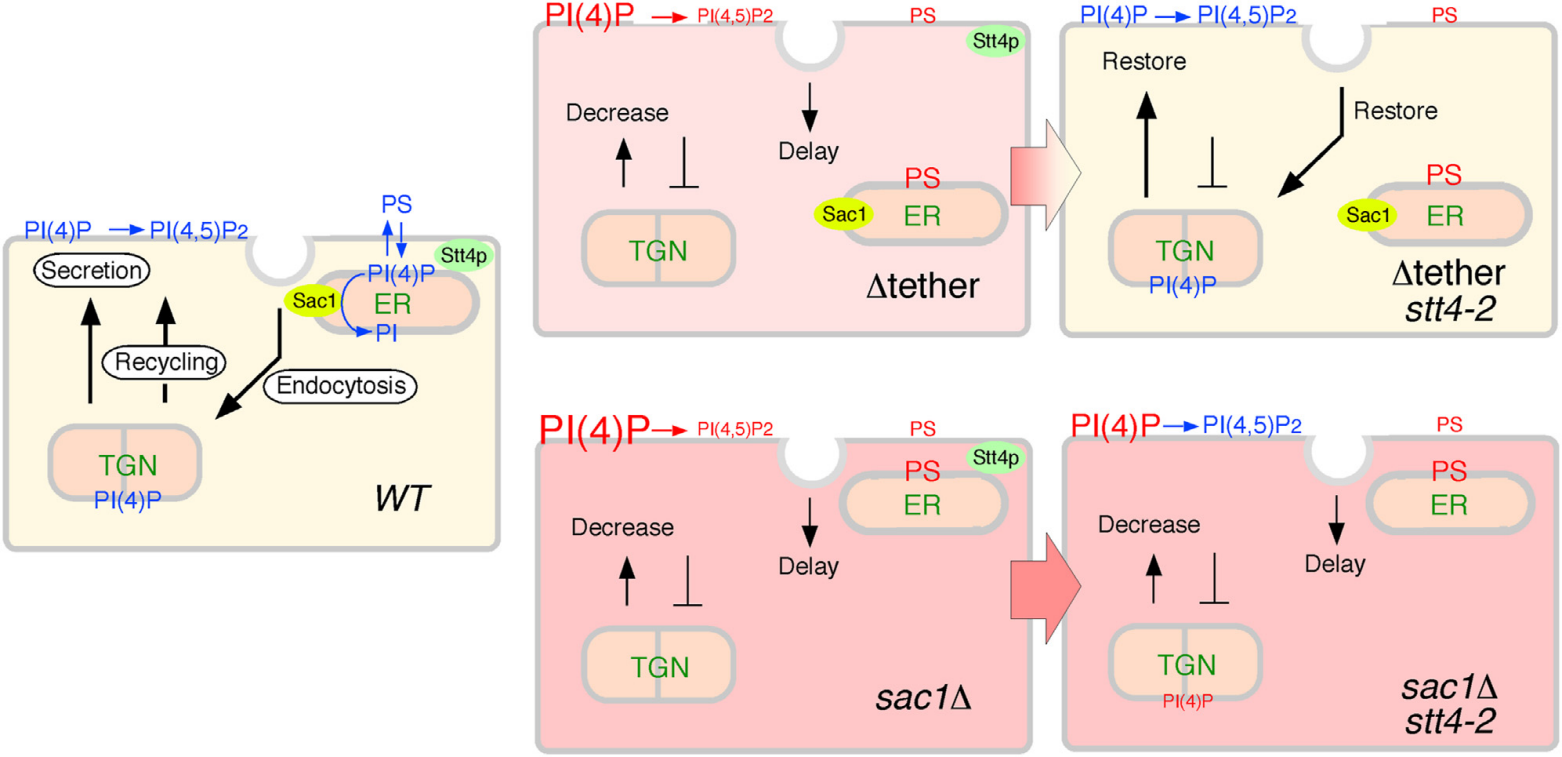
**The relationship between anionic phospholipid levels at the PM and intracellular vesicular transport**. Sac1 localizes at the ER membrane and regulates PM PtdIns(4)P levels. In the Δtether mutant, the PM PtdIns(4)P level increased, PS accumulates to the intracellular compartments, and endocytosis, secretion, and recycling pathways are defective. In the *sac1*Δ mutant, the PM PtdIns(4)P level further increased, and each transport pathway is also defective, with a particularly large defect in the recycling pathway. Inactivation of Stt4 restores the PM PtdIns(4)P level to wild-type level in the Δtether mutant and partially restores it in the *sac1*Δ mutant, but does not restore aberrant PS localization. Furthermore, inactivation of Stt4 restores the endocytosis and secretory pathways in the Δtether mutant but does not restore either pathway in the *sac1*Δ mutant. Similar to PS localization, the recycling pathway is not restored by the inactivation of Stt4 in either mutant. The *black* T bars represent the step that indicates transport inhibition.

It was originally believed that Sac1 functions in *trans* at the ER-PM contact sites (15), but later studies suggested that Sac1 acts in *cis* after PtdIns(4)P has been transferred to the ER (51). ^3^H-inositol-labeling of PtdIns has shown that both the Δtether and *sac1*Δ mutants exhibit increased PtdIns(4)P levels relative to wild-type cells, although no significant difference in the increased level of PtdIns(4)P was observed between the two mutants (15, 16, 35). It is also reported that Stt4 produces PtdIns(4)P that accumulates in *sac1* ts mutants (35) and that introduction of *stt4* ts mutation into the *sac1*Δ mutant restores aberrant PtdIns(4)P distribution at ER and vacuolar membrane in the *sac1*Δ mutant (33). In contrast, we showed that the *sac1*Δ and Δtether mutants exhibited increased PtdIns(4)P levels at the PM, and that the PtdIns(4)P level in the *sac1*Δ mutant was increased about 2-fold relative to the Δtether mutant. Additionally, we demonstrated that the increased PtdIns(4)P level at the PM in the *sac1*Δ mutant was decreased to around half in the *stt4-2 sac1*Δ double mutant, but the level was ∼2.3 fold higher than that in wild-type cells. These discrepancies may have been due to the fact that [^3^H]inositol labeling measures the amount of PtdIns(4)P in the whole cell, whereas in the present study only the amount of PtdIns(4)P in the PM was measured. Since the *sac1*Δ mutant has a more severe defect of endocytic internalization, PtdIns(4)P might accumulate more at the PM. Endocytic internalization was defective in both the Δtether and *sac*1Δ mutants, but Stt4 inactivation did not normalize the defect in the *sac1*Δ mutant, even though the PtdIns (4,5)P_2_ levels in both were recovered by Stt4 inactivation (Fig. 6). Since it is known that PtdIns(4)P plays essential roles in regulation of vesicle trafficking at the Golgi (11), this result may suggest the importance of PtdIns(4)P in endocytic internalization. Deletion of the *SAC1* gene also disrupts vacuole morphology, and this defect can be abrogated by Stt4 deletion, or by overexpression of Inp52 or Inp53 (35), suggesting that PtdIns(4)P homeostasis is also crucial for vacuolar fusion and/or transport.

A previous study has reported transient interaction between Osh2 and nascent endocytic sites on the PM near cER rims (20). Through its interactions with Scs2 and endocytic type I myosin Myo5, it has been proposed that Osh2 regulates cER-endocytic site associations to promote actin patch assembly for endocytic internalization (21). Disruption of cER-endocytic site associations in the *scs2*Δ *scs22*Δ mutant increases the lifetimes of Sla1 and Abp1 patches and interferes with vesicle scission (20). We demonstrated here that the lifetimes of Abp1 patches were extended in the Δtether mutant, and that the mutant showed significant delay in the uptake and transport of endocytic cargo into the cell. Interestingly, although the cER-PM contact sites remained disrupted, these endocytic defects were normalized by inactivation of Stt4. Therefore, our results suggest that the endocytic defects observed in the Δtether mutant might not be due to disruption of contact between the cER and the endocytic site. This possibility is supported by the finding that the endocytic defect in the *sac1*Δ mutant was not abrogated by Stt4 inactivation. The *sac1*Δ mutant showed endocytic defects similar to those of the Δtether mutant, but more severe, although contact between the cER and the PM was not disrupted (40). Similar to the *osh2*Δ *osh3*Δ mutant (20), actin polymerization at endocytic sites was delayed in the *sac1*Δ mutant, and this delay was not abrogated by Stt4 inactivation. Previous studies have shown that sterol is required for the internalization step of endocytosis (52, 53) and that Osh2-dependent sterol transfer is important for actin polymerization at endocytic sites (20, 21). Osh2 functions as a sterol/PtdIns(4)P exchanger at ER–PM contact sites, and constant synthesis and hydrolysis of PtdIns(4)P drives the continuous exchange cycles (54, 55). Thus, in the *sac1*Δ mutant with defects in PtdIns(4)P hydrolysis, sterols are not properly transported to the endocytosis sites, and actin polymerization might be delayed.

We showed that in the Δtether mutant the decreased secretion of secNluc was normalized by Stt4 inactivation but defective recycling of GFP-Snc1 was not restored (Fig. 6). In both yeast and mammalian cells, PtdIns(4)P is a key regulator of the secretory pathway (56). PtdIns(4)P generated in the Golgi is required for the recruitment of clathrin adaptor proteins to the TGN and the subsequent formation and budding of transport vesicles (57, 58, 59). A reduction of PtdIns(4)P at the Golgi in the *pik1* mutant strongly inhibits anterograde transport from the Golgi (24, 60, 61). In contrast, an increased PtdIns(4)P level also leads to a defect of secretory vesicle transport to the PM (62, 63). Therefore, it is likely that proper maintenance of the PtdIns(4)P level at the Golgi plays a crucial role in the secretory pathway. On the other hand, PS is concentrated in the recycling pathway and involved in retrograde transport from the endosome (64). Thus, it is possible that the secretory and recycling pathways are differently regulated by PtdIns(4)P and PS. The levels of PtdIns(4)P and PS at the PM are significantly affected in Δtether and *sac1*Δ mutants, with inactivation of Stt4 restoring PtdIns(4)P levels but not intracellular accumulation of PS (39), suggesting that proper localization of PS is not simply regulated by PtdIns(4)P levels, but probably requires continuous turnover of PtdIns(4)P by PI 4-kinase and phosphatase.

## Experimental procedures

### Yeast strains, growth conditions, and plasmids

The yeast strains used in this study are listed in Table S1. All strains were grown in standard rich medium (YPD) or synthetic medium (SM) supplemented with 2% glucose and appropriate amino acids. C-terminal GFP or mCherry tagging of proteins was performed as described previously (65). The *stt4-2* mutant was generated as follows: The *Not*I-*Sac*II fragment, which contains the *Saccharomyces cerevisiae ADH1* terminator and the *URA3MX6* module, was amplified by PCR, and inserted into *Not*I- and *Sac*II-digested pBluescript II SK (pBS-TADH-URA3). To create a plasmid library containing *STT4* gene fragments (nt 4428–5700) carrying various mutations, error-prone PCR products amplified by JT2057 and JT2058, using yeast genome DNA as a template, were digested with BamHI and NotI and inserted into the BamHI and NotI-digested pBS-TADH-URA3 (12). To integrate the plasmid library at the endogenous locus of the *STT4* gene, the plasmid was linearized by NruI and transformed into wild-type cells, and transformants were subsequently grown on SD-URA plates at 25 ^o^C. After 3 to 4 days, ∼1000 transformants were replica-plated on SD-URA plates and subsequently grown on at 25 or 37 ^o^C for 2 to 3 days.

### Fluorescence microscopy

Fluorescence microscopy was performed using an Olympus IX83 microscope equipped with a x100/NA 1.40 (Olympus) or a x100/NA 1.49 (Olympus) objective and Orca-R2 cooled CCD camera (Hamamatsu), using Metamorph software (Universal Imaging). For TIRF illumination, optically pumped semiconductor laser (OPSL) (Coherent) with emission of at 488 nm (OBIS 488LS-50) and at 561 nm (OBIS 561LS-50) were used to excite GFP or mCherry/Alexa594, respectively. Simultaneous imaging of red and green fluorescence was performed using an Olympus IX83 microscope, described above, and an image splitter (Dual-View; Optical Insights) that divided the red and green components of the images with a 565-nm dichroic mirror and passed the red component through a 630/50 nm filter and the green component through a 530/30 nm filter. Simultaneous imaging of red and green fluorescence was performed using an Olympus IX83 microscope, described above, and an image splitter (Dual-View; Optical Insights) that divided the red and green components of the images with a 565-nm dichroic mirror and passed the red component through a 630/50 nm filter and the green component through a 530/30 nm filter. These split signals were taken simultaneously with one CCD camera, described above. All cells were imaged during the early-to mid-logarithmic phase. Images for analysis of co-localization of red and green signals were acquired using simultaneous imaging (64.5 nm pixel size), described above.

### Fluorescence labeling of *α*-factor and endocytosis assays

Fluorescence labeling of α-factor was performed as described previously (36). For endocytosis assays, cells were grown to an OD600 of ∼0.5 in 0.5 ml YPD, briefly centrifuged, and resuspended in 20 μl SM with 5 μM Alexa Fluor-labeled α-factor. After incubation on ice for 2 h, the cells were washed with ice-cold SM. Internalization was initiated by addition of SM containing 4% glucose and amino acids at 25 ^o^C.

### ^35^S-labeled *α*-factor internalization and binding assay

Preparation and internalization of ^35^S-labeled α-factor was performed as described previously (66). For the binding assay, cells were grown to an OD600 of ∼0.3 in 1 ml YPD at 25 ^o^C, briefly centrifuged, and resuspended in 50 μl SM with 1% (w/v) BSA and ^35^S-labeled α-factor on ice. After incubation on ice for 2 h, cells were washed with ice-cold SM and measured for their radioactivity.

### Nanoluc luciferase-based secretion assay

The secNluc reporter was expressed as described previously (39). To integrate the secNluc reporter plasmid (pRS305-P*TPI*-secNluc-T*TPI*) at the LEU2 locus, the plasmid was linearized by EcoRI and transformed into wild-type or mutant cells. For the secretion assay, cells expressing secNluc reporter were grown to an OD600 of ∼0.5 in 1.0 ml YPD, briefly centrifuged, and resuspended in fresh 800 μl YPD. After incubation at 25 ^o^C for 30 min, 100 μl of the culture medium was aliquoted and centrifuged, and luciferase activities in the supernatants were measured by Nano-Glo luciferase assay system (Promega, Madison, WI).

## Data availability

All data are contained within the manuscript.

## Supporting information

This article contains supporting information.

## Conflict of interest

The authors declare that they have no conflict of interest with the content of this article.

## Figures and Tables

**Figure S2:**