# Supplementary material for: Maintenance of proper phosphatidylinositol-4-phosphate level by Stt4 and Sac1 contributes to vesicular transport to and from the plasma membrane
Source: J Biol Chem. 2025 Jun 21;301(8):110410. doi: 10.1016/j.jbc.2025.110410 (PMC12303058; doi:10.1016/j.jbc.2025.110410)
Supplement: Table S1 [file mmc1.docx]

**Table S1**. Yeast Strains used in this study

Strain Genotype Source

JJTY0501 *Mat***a** *his3*Δ*1* *leu2*Δ*0 ura3*Δ*0* *lys2*Δ*0 bar1*Δ::*LEU2* Toshima lab

JJTY1219 *Mat***a** *his3*Δ*1* *leu2*Δ*0* *ura3*Δ*0* *lys2*Δ*0* Toshima lab

JJTY3492 *Mat***a** *his3*-Δ*200* *leu2-3*, *112* *ura3-52* *lys2-801 SLA1-GFP*::*HIS3 ABP1-mCherry*::*LEU2* Toshima lab

JJTY5669 *Mat***a** *his3*Δ*1* *leu2*Δ*0* *ura3*Δ*0* *lys2*Δ*0 GFP-STT4*::*HIS3 (ZWF1 promoter) SLA1-mCherry*::*URA3* This study

JJTY5897 *Mat***a** *his3*Δ*1* *leu2*Δ*0 ura3*Δ*0* *lys2*Δ*0 stt4*(*F1777S*)::*URA3* This study

JJTY7265 *Mat***a** *his3*Δ*1* *leu2*Δ*0 ura3*Δ*0* *lys2*Δ*0* *GFP-2PH^OSH2^*::*HIS3 ABP1-mCherry*::*URA3* This study

JJTY7266 *Mat***a** *his3*Δ*1* *leu2*Δ*0 ura3*Δ*0* *lys2*Δ*0 GFP-2PH^PLCδ^*::*HIS3 ABP1-mCherry*::*URA3* This study

JJTY7636 *Mat***a** *his3*Δ*1* *leu2*Δ*0 ura3*Δ*0* *lys2*Δ*0 sac1*Δ::*KanMX6 GFP-2PH^PLCδ^*::*HIS3* This study

JJTY7637 *Mat***a** *his3*Δ*1* *leu2*Δ*0 ura3*Δ*0* *lys2*Δ*0 sac1*Δ::*KanMX6 GFP-2PH^OSH2^*::*HIS3* This study

JJTY8864 *Mat***a** *his3*Δ*1* *leu2*Δ*0* *ura3*Δ*0* *lys2*Δ*0* *stt4*(*S1747P*)::*URA3* This study

JJTY8865 *Mat***a** *his3*Δ*1* *leu2*Δ*0* *ura3*Δ*0* *lys2*Δ*0* *stt4*(*S1747P*)::*URA3 bar1*Δ::*LEU2* This study

JJTY8866 *Mat***a** *his3*Δ*1* *leu2*Δ*0* *ura3*Δ*0* *lys2*Δ*0* *stt4*(*S1747P*)::*URA3 GFP-2PH^OSH2^*::*HIS3* This study

JJTY8869 *Mat***a** *his3*Δ*1* *leu2*Δ*0* *ura3*Δ*0* *lys2*Δ*0* *stt4*(*S1747P*)::*URA3 GFP-2PH^PLCδ^*::*HIS3* This study

JJTY8874 *Mat***a** *his3*Δ*1* *leu2*Δ*0* *ura3*Δ*0* *lys2*Δ*0* *stt4*(*S1747P*)::*URA3 SLA1-GFP*::*HIS3 ABP1-mCherry*::*LEU2* This study

JJTY9162 *Mat***a** *his3*Δ*1* *leu2*Δ*0* *ura3*Δ*0* *lys2*Δ*0* *tcb1*Δ::*KanMX6 tcb2*Δ::*KanMX6 tcb3*Δ::*KanMX6 ist2*Δ::*KanMX6*

*scs2*Δ::*HphMX4* *scs22*Δ::*NatMX4 bar1*Δ::*LEU2* This study

JJTY9359 *Mat***a** *his3*Δ*1* *leu2*Δ*0* *ura3*Δ*0* *lys2*Δ*0* *tcb1*Δ::*KanMX6 tcb2*Δ::*KanMX6 tcb3*Δ::*KanMX6 ist2*Δ::*KanMX6*

*scs2*Δ::*HphMX4* *scs22*Δ::*NatMX4* *SLA1-GFP*::*HIS3 ABP1-mCherry*::*LEU2* This study

JJTY9362 *Mat***a** *his3*Δ*1* *leu2*Δ*0* *ura3*Δ*0* *lys2*Δ*0* *stt4*(*S1747P*)::*URA3* *tcb1*Δ::*KanMX6* *tcb2*Δ::*KanMX6*

*tcb3*Δ::*KanMX6* *ist2*Δ::*KanMX6* *scs2*Δ::*HphMX4* *scs22*Δ::*NatMX4 bar1*Δ::*LEU2* This study

JJTY9363 *Mat***a** *his3*Δ*1* *leu2*Δ*0* *ura3*Δ*0* *lys2*Δ*0* *tcb1*Δ::*KanMX6 tcb2*Δ::*KanMX6 tcb3*Δ::*KanMX6*

*ist2*Δ::*KanMX6 scs2*Δ::*HphMX4* *scs22*Δ::*NatMX4 GFP-2PH^OSH2^*::*HIS3* This study

JJTY9364 *Mat***a** *his3*Δ*1* *leu2*Δ*0* *ura3*Δ*0* *lys2*Δ*0* *tcb1*Δ::*KanMX6 tcb2*Δ::*KanMX6 tcb3*Δ::*KanMX6*

*ist2*Δ::*KanMX6 scs2*Δ::*HphMX4* *scs22*Δ::*NatMX4 GFP-2PH^PLCδ^*::*HIS3* This study

JJTY9365 *Mat***a** *his3*Δ*1* *leu2*Δ*0* *ura3*Δ*0* *lys2*Δ*0* *stt4S1747P*::*URA3* *tcb1*Δ::*KanMX6* *tcb2*Δ::*KanMX6*

*tcb3*Δ::*KanMX6* *ist2*Δ::*KanMX6* *scs2*Δ::*HphMX4* *scs22*Δ::*NatMX4 GFP-2PH^OSH2^*::*HIS3* This study

JJTY9366 *Mat***a** *his3*Δ*1* *leu2*Δ*0* *ura3*Δ*0* *lys2*Δ*0* *stt4*(*S1747P*)::*URA3* *tcb1*Δ::*KanMX6* *tcb2*Δ::*KanMX6 tcb3*Δ::*KanMX6*

*ist2*Δ::*KanMX6* *scs2*Δ::*HphMX4* *scs22*Δ::*NatMX4 GFP-2PH^PLCδ^*::*HIS3* This study

JJTY9504 *Mat***a** *his3*Δ*1* *leu2*Δ*0 ura3*Δ*0* *lys2*Δ*0 sac1*Δ::*KanMX6 SLA1-GFP*::*HIS3 ABP1-mCherry*::*LEU2* This study

JJTY9616 *Mat***a** *his3*Δ*1* *leu2*Δ*0* *ura3*Δ*0* *lys2*Δ*0 GFP-MSS4*::*HIS3 (ZWF1 promoter) PIL1-mCherry*::*LEU2* This study

JJTY9617 *Mat***a** *his3*Δ*1* *leu2*Δ*0* *ura3*Δ*0* *lys2*Δ*0* *tcb1*Δ::*KanMX6 tcb2*Δ::*KanMX6 tcb3*Δ::*KanMX6 ist2*Δ::*KanMX6*

*scs2*Δ::*HphMX4* *scs22*Δ::*NatMX4 GFP-MSS4*::*HIS3 (ZWF1 promoter) PIL1-mCherry*::*LEU2* This study

JJTY9964 *Mat***a** *his3*Δ*1* *leu2*Δ*0* *ura3*Δ*0* *lys2*Δ*0 GFP-STT4*::*HIS3* (*ZWF1 promoter*)[pRS316*-*P*TPI1*-*CTS1 SS-mCherry-HDEL*] This study

JJTY9968 *Mat***a** *his3*Δ*1* *leu2*Δ*0* *ura3*Δ*0* *lys2*Δ*0* *tcb1*Δ::*KanMX6 tcb2*Δ::*KanMX6 tcb3*Δ::*KanMX6 ist2*Δ::*KanMX6*

*scs2*Δ::*HphMX4* *scs22*Δ::*NatMX4 GFP-STT4*::*HIS3* (*ZWF1 promoter*) [pRS316*-*P*TPI1*-*CTS1 SS-mCherry-HDEL*] This study

JTY9969 *Mat***a** *his3*Δ*1* *leu2*Δ*0* *ura3*Δ*0* *lys2*Δ*0 GFP-STT4*::*HIS3 (ZWF1 promoter) TCB1-mCherry*::*LEU2* This study

JTY9970 *Mat***a** *his3*Δ*1* *leu2*Δ*0* *ura3*Δ*0* *lys2*Δ*0 GFP-STT4*::*HIS3 (ZWF1 promoter) TCB2-mCherry*::*LEU2* This study

JJTY9971 *Mat***a** *his3*Δ*1* *leu2*Δ*0* *ura3*Δ*0* *lys2*Δ*0 GFP-STT4*::*HIS3 (ZWF1 promoter) TCB3-mCherry*::*LEU2* This study

JJTY9972 *Mat***a** *his3*Δ*1* *leu2*Δ*0* *ura3*Δ*0* *lys2*Δ*0 GFP-STT4*::*HIS3 (ZWF1 promoter) IST2-mCherry*::*LEU2* This study

JJTY9973 *Mat***a** *his3*Δ*1* *leu2*Δ*0* *ura3*Δ*0* *lys2*Δ*0 GFP-STT4*::*HIS3 (ZWF1 promoter) SCS2-mCherry*::*LEU2* This study

JJTY9980 *Mat***a** *his3*Δ*1* *leu2*Δ*0 ura3*Δ*0* *lys2*Δ*0 sac1*Δ::*KanMX6 bar1*Δ::*LEU2* This study

JJTY9982 *Mat***a** *his3*Δ*1* *leu2*Δ*0* *ura3*Δ*0* *lys2*Δ*0 bar1Δ*::*HphMX4 SLA1-GFP*::*HIS3* This study

JJTY9983 *Mat***a** *his3*Δ*1* *leu2*Δ*0 ura3*Δ*0* *lys2*Δ*0 stt4*(*S1747P*)::*URA3 sac1*Δ::*KanMX6 bar1*Δ::*LEU2* This study

JJTY9985 *Mat***a** *his3*Δ*1* *leu2*Δ*0 ura3*Δ*0* *lys2*Δ*0 stt4*(*S1747P*)::*URA3 sac1*Δ::*KanMX6 GFP-2PH^OSH2^*::*HIS3* This study

JJTY9986 *Mat***a** *his3*Δ*1* *leu2*Δ*0 ura3*Δ*0* *lys2*Δ*0 stt4*(*S1747P*)::*URA3 sac1*Δ::*KanMX6 GFP-2PH^PLCδ^*::*HIS3* This study

JJTY10121 *Mat***a** *his3*Δ*1* *leu2*Δ*0* *ura3*Δ*0* *lys2*Δ*0* *stt4*(*S1747P*)::*URA3* *tcb1*Δ::*KanMX6* *tcb2*Δ::*KanMX6 tcb3*Δ::*KanMX6*

*ist2*Δ::*KanMX6* *scs2*Δ::*HphMX4* *scs22*Δ::*NatMX4 SLA1-GFP*::*HIS3 ABP1-mCherry*::*LEU2* This study

JJTY10122 *Mat***a** *his3*Δ*1* *leu2*Δ*0* *ura3*Δ*0* *lys2*Δ*0* *tcb1*Δ::*KanMX6 tcb2*Δ::*KanMX6 tcb3*Δ::*KanMX6 ist2*Δ::*KanMX6*

*scs2*Δ::*HphMX4* *scs22*Δ::*NatMX4 bar1*Δ::*LEU2 SLA1-GFP*::*HIS3* This study

JJTY10147 *Mat***a** *his3*Δ*1* *leu2*Δ*0 ura3*Δ*0* *lys2*Δ*0 stt4*(*S1747P*)::*URA3 sac1*Δ::*KanMX6 SLA1-GFP*::*HIS3 ABP1-mCherry*::*LEU2* This study

JJTY10962 *Mat***a** *his3*Δ*1* *leu2*Δ*0* *ura3*Δ*0* *lys2*Δ*0* [*pSivh-*P*ste2-GFP-SNC1*] This study

JJTY10963 *Mat***a** *his3*Δ*1* *leu2*Δ*0* *ura3*Δ*0* *lys2*Δ*0 tcb1*Δ::*KanMX6 tcb2*Δ::*KanMX6 tcb3*Δ::*KanMX6 ist2*Δ::*KanMX6*

*scs2*Δ::*HphMX4 scs22*Δ::*NatMX4* [*pSivh-*P*ste2-GFP-SNC1*] This study

JJTY11465 *Mat***a** *his3*Δ*1* *leu2*Δ*0* *ura3*Δ*0* *lys2*Δ*0* [*pSivh-*P*ste2-GFP-Lact-C2*] This study

JJTY11466 *Mat***a** *his3*Δ*1* *leu2*Δ*0* *ura3*Δ*0* *lys2*Δ*0 tcb1*Δ::*KanMX6 tcb2*Δ::*KanMX6 tcb3*Δ::*KanMX6 ist2*Δ::*KanMX6*

*scs2*Δ::*HphMX4 scs22*Δ::*NatMX4* [*pSivh-*P*ste2-GFP-Lact-C2*] This study

JJTY11481 *Mat***a** *his3*Δ*1* *leu2*Δ*0* *ura3*Δ*0* *lys2*Δ*0* [*pSivh-*P*TPI1-MFA1ss-Nluc-6×His*] This study

JJTY12287 *Mat***a** *his3*Δ*l leu2Δ0 ura3Δ0 met15Δ0 sac1Δ*::*KanMX6* [*pSivh-*P*ste2-GFP-SNC1*] This study

JJTY12288 *Mat***a** *his3*Δ*1* *leu2*Δ*0 ura3*Δ*0* *lys2*Δ*0 stt4S1747P* ::*URA3 sac11*Δ*::KanMX6* [*pSivh-*P*ste2-GFP-SNC1*] This study

JJTY12289 *Mat***a** *his3*Δ*l leu2Δ0 ura3Δ0 met15Δ0 sac1Δ*::*KanMX6* [*pSivh-*P*ste2-GFP-Lact-C2*] This study

JJTY12290 *Mat***a** *his3*Δ*1* *leu2*Δ*0 ura3*Δ*0* *lys2*Δ*0 stt4*(*S1747P*)::*URA3 sac1*Δ*::KanMX6* [*pSivh-*P*ste2-GFP-Lact-C2*] This study

JJTY13673 *Mat***a** *his3*Δ*1* *leu2*Δ*0 ura3*Δ*0* *lys2*Δ*0 stt4*(*S1747P*)::*URA3* [*pSivh-*P*TPI1-MFA1ss-Nluc-6×His*]

JJTY13674 *Mat***a** *his3*Δ*1* *leu2*Δ*0* *ura3*Δ*0* *lys2*Δ*0 tcb1*Δ::*KanMX6 tcb2*Δ::*KanMX6 tcb3*Δ::*KanMX6 ist2*Δ::*KanMX6*

*scs2*Δ::*HphMX4 scs22*Δ::*NatMX4* [*pSivh-*P*TPI1-MFA1ss-Nluc-6×His*] This study

JJTY13675 *Mat***a** *his3*Δ*1* *leu2*Δ*0 ura3*Δ*0* *lys2*Δ*0 stt4*(*S1747P*)::*URA3 sac1*Δ*::KanMX6 tcb1*Δ::*KanMX6 tcb2*Δ::*KanMX6*

*tcb3*Δ::*KanMX6 ist2*Δ::*KanMX6 scs2*Δ::*HphMX4 scs22*Δ::*NatMX4* [*pSivh-*P*TPI1-MFA1ss-Nluc-6×His*] This study

JJTY13676 *Mat***a** *his3*Δ*l leu2Δ0 ura3Δ0 met15Δ0 sac1Δ*::*KanMX6* [*pSivh-*P*TPI1-MFA1ss-Nluc-6×His*] This study

JJTY13677 *Mat***a** *his3*Δ*1* *leu2*Δ*0 ura3*Δ*0* *lys2*Δ*0 stt4*(*S1747P*)::*URA3 sac1*Δ*::KanMX6* [*pSivh-*P*TPI1-MFA1ss-Nluc-6×His*] This study

JJTY13678 *Mat***a** *his3*Δ*1* *leu2*Δ*0 ura3*Δ*0* *lys2*Δ*0 stt4*(*S1747P*)::*URA3* [*pSivh-*P*ste2-GFP-SNC1*] This study

JJTY13679 *Mat***a** *his3*Δ*1* *leu2*Δ*0* *ura3*Δ*0* *lys2*Δ*0 stt4*(*S1747P*)::*URA3 tcb1*Δ::*KanMX6 tcb2*Δ::*KanMX6 tcb3*Δ::*KanMX6*

*ist2*Δ::*KanMX6 scs2*Δ::*HphMX4 scs22*Δ::*NatMX4* [*pSivh-*P*ste2-GFP-SNC1*] This study

JJTY13680 *Mat***a** *his3*Δ*1* *leu2*Δ*0 ura3*Δ*0* *lys2*Δ*0 stt4*(*S1747P*)::*URA3* [*pSivh-*P*ste2-GFP-Lact-C2*] This study

JJTY13681 *Mat***a** *his3*Δ*1* *leu2*Δ*0 ura3*Δ*0* *lys2*Δ*0 stt4*(*S1747P*)::*URA3 tcb1*Δ::*KanMX6 tcb2*Δ::*KanMX6 tcb3*Δ::*KanMX6*

*ist2*Δ::*KanMX6 scs2*Δ::*HphMX4 scs22*Δ::*NatMX4* [*pSivh-*P*ste2-GFP-Lact-C2*] This study

JTY14117 *Mat***a** *his3*Δ*1 leu2*Δ*0 ura3*Δ*0 lys2*Δ*0 GFP-STT*::*HIS3 (Zwf1 pro) SEC63-mCherry*::*URA3* This study

JTY13985 *Mat***a** *his3*Δ*1* *leu2*Δ*0* *ura3*Δ*0* *lys2*Δ*0 GFP-STT4*::*HIS3 (ZWF1 promoter) mTurquoise2-SSO1*::*LEU2 (SIVl:BglII)*

*SCS2-mCherry*::*URA3* This study

JTY13986 *Mat***a** *his3*Δ*1* *leu2*Δ*0* *ura3*Δ*0* *lys2*Δ*0 GFP-STT4*::*HIS3 (ZWF1 promoter) mTurquoise2-SSO1*::*LEU2 (SIVl:BglII)*

*IST2-mCherry*::*URA3* This study

JTY13990 *Mat*a *his3*Δ*1* *leu2*Δ*0 ura3*Δ*0* *lys2*Δ*0 tcb1*Δ::*KanMX6 tcb2*Δ::*KanMX6 tcb3*Δ::*KanMX6 ist2*Δ::*KanMX6*

*scs2*Δ::*HphMX4* *scs22*Δ::*NatMX4 sac1*Δ::*LEU2 GFP-2PH^PLCδ^*::*HIS3* This study

JTY13991 *Mat*a *his3*Δ*1* *leu2*Δ*0 ura3*Δ*0* *lys2*Δ*0 tcb1*Δ::*KanMX6 tcb2*Δ::*KanMX6 tcb3*Δ::*KanMX6 ist2*Δ::*KanMX6*

*scs2*Δ::*HphMX4* *scs22*Δ::*NatMX4 sac1*Δ::*LEU2* [*pSivh-*P*ste2-GFP-Lact-C2*] This study

JTY13992 *Mat***a** *his3*Δ*1* *leu2*Δ*0* *ura3*Δ*0* *lys2*Δ*0 ist2*Δ::*Kan GFP-STT4(ZWF promoter)*::*HIS3 SCS2-mCherry*::*URA3* This study

JTY13994 *Mat***a** *his3*Δ*1* *leu2*Δ*0* *ura3*Δ*0* *lys2*Δ*0 GFP-STT4*::*HIS3 (ZWF1 promoter) IST2-mCherry*::*LEU2 scs2*Δ::*URA3*

*scs22*Δ::*HphMX4* This study

JTY13997 *Mat***a** *his3*Δ*1* *leu2*Δ*0 ura3*Δ*0* *lys2*Δ*0 stt4S1747P*::*URA3 SCS2-GFP*::*HIS3 k-his3*Δ::*LEU2*

[pRS306*-*P*TPI1*-*CTS1 SS-mCherry-HDEL*] This study
